# Multi-Locus Phylogeny and Morphology Reveal Two New Species of *Hypoxylon* (Hypoxylaceae, Xylariales) from Motuo, China

**DOI:** 10.3390/microorganisms12010072

**Published:** 2023-12-29

**Authors:** An-Hong Zhu, Zi-Kun Song, Jun-Fang Wang, Hao-Wen Guan, Hai-Xia Ma

**Affiliations:** 1 Institute of Tropical Bioscience and Biotechnology, Chinese Academy of Tropical Agricultural Sciences, Hainan Institute for Tropical Agricultural Resources, Haikou 571101, China; 18289679317@163.com (A.-H.Z.); michellesong2021@yeah.net (Z.-K.S.); 15379730137@163.com (J.-F.W.); 17725357096@163.com (H.-W.G.); 2 School of Ecology and Nature Conservation, Beijing Forestry University, Beijing 100083, China; 3 Rubber Research Institute, Chinese Academy of Tropical Agricultural Sciences, Haikou 571101, China; 4 College of Plant Protection, Jilin Agricultural University, Changchun 130118, China; 5 School of Life Science, Liaoning University, Shenyang 110036, China; 6 Haikou Key Laboratory for Protection and Utilization of Edible and Medicinal Fungi, Haikou 571101, China; 7 Hainan Key Laboratory of Tropical Microbe Resources, Haikou 571101, China

**Keywords:** Ascomycota, multigene phylogeny, new species, taxonomy, Xylariales

## Abstract

Hypoxylaceous fungi are abundant in China, but their discovery and report are uneven in various provinces, with more fungi in Yunnan and Hainan and fewer fungi in Tibet. During the investigation of macro-fungi in Motuo county, Tibet Autonomous Region, we collected a number of xylarialean specimens. Six hypoxylaceous specimens growing on dead angiosperm were collected from the forests of Motuo county, and they were described and illustrated as two new species in *Hypoxylon* based on a combination of morphological characters and molecular evidence. *Hypoxylon diperithecium* was characterized by its bistratal perithecia, purple-brown stromatal granules, citrine to rust KOH-extractable pigments, and light brown to brown ascospores ellipsoid-inequilateral with conspicuous coil-like ornamentation. *Hypoxylon tibeticum* was distinct from other species by having pulvinate and applanate stromata, surface vinaceous, with orange granules, orange KOH-extractable pigments, and brown ascospores with inconspicuous ornamentation. The multi-gene phylogenetic analyses (ITS-LSU-RPB2-TUB) supported the two new taxa as separate lineages in the genus *Hypoxylon*. A key to all known *Hypoxylon* taxa from China is provided.

## 1. Introduction

Motuo county, between 27°33′–29°55′ N and 93°45′–96°05′ E, is located in the southeastern Tibet Autonomous Region of southwestern China, and it covers an area of 34,000 square kilometers [1,2,3]. The area enjoys the tropical monsoon rainforest and subtropical humid monsoon climate and is one of the most abundant regions of light, heat and water [4,5]. Its complex topography and diverse habitat abound with different kinds of biological resources, and the area has long been reputed as the “world’s biological gene bank”. There are extremely abundant animal and plant resources, and more than 3000 plant species, 850 genera and 230 families have been reported in the county (http://www.motuo.gov.cn/, accessed on 18 September 2023) [6,7,8,9,10]. Due to severe climatic conditions and inconvenient transportation, few investigations and studies of macro-fungi diversity have been carried out in Motuo county. In the past, about 200 species of macro-fungi have been reported in Motuo county [11,12,13,14,15,16,17,18], among which four species are pyrenomycetous fungi [12]. In recent years, some new species and new records of pyrenomycetous fungi have been discovered in the area, e.g., *Eutypella motuoensis* Hai X. Ma & Z.E. Yang, *Hypoxylon damuense* Hai X. Ma, Z.K. Song & Y. Li, *H. medogense* Hai X. Ma, Z.K. Song & Y. Li, *H. zangii* Hai X. Ma, Z.K. Song & Y. Li, *Annulohypoxylon leptascum* (Speg.) Y.M. Ju, J.D. Rogers & H.M. Hsieh, *Daldinia bambusicola* Y.M. Ju, J.D. Rogers & F. San Martín, *H. sublenormandii* Suwann., Rodtong, Thienh. & Whalley, and so on [19,20,21].

In order to further understand the diversity of macro-fungi in Motuo county, we carried out a field survey focusing on xylarialean fungi in September 2021. We collected a number of xylarialean specimens, including *Annulohypoxylon*, *Daldinia*, *Diatrype*, *Eutypella*, *Neoeutypella*, *Hypoxylon*, *Jackrogersella*, and *Xylaria*. *Hypoxylon* Bull. (Hypoxylaceae, Ascomycota) was established by Bulliard in 1791 and typified with *H. fragiforme* (Pers.) J. Kickx f. [22,23]. The type genus is the largest genera in the family Hypoxylaceae, with more than 200 species accepted [24,25,26] and 1188 epithets in the Index Fungorum (http://www.indexfungorum.org/Names/Names.asp, accessed on 22 September 2023). Most taxa of the genus are mainly associated with angiosperm wood as saprotrophs and endophytes, and degrade cellulose and lignin, which play a key role in the substance circulation of a forest ecosystem [24,27,28,29,30,31]. Currently, the placement of *Hypoxylon* and related genera in Hypoxylaceae is confusing because many are polyphyletic [32,33,34,35,36]. In order to further understand the species diversity and phylogeny of Hypoxylaceae, we carried out complete morphological and multi-gene phylogenetic studies on these specimens from Motuo county. In this study, two new species are introduced based on morphological and phylogenetic evidence.

## 2. Materials and Methods

### 2.1. Morphological Studies

The studied specimens were deposited at the Fungarium of the Institute of Tropical Bioscience and Biotechnology, Chinese Academy of Tropical Agricultural Sciences (FCATAS). Morphological observations and measurements in this study followed Ma et al. (2018) [24] and Song et al. (2022) [37]. The morphology of stromata and perithecia were observed and measured by a VHX-6000 microscope (Osaka, Japan). Microscopic characteristics, measurements and photographs of the teleomorph were made from slide preparations of fresh stromata mounted in water, 10% KOH and Melzer’s reagent. Sections were observed at a magnification up to ×1000 by using an Olympus IX73 inverted fluorescence microscope (Olympus, Tokyo, Japan). The ornamentation of ascospores were observed with a scanning electron microscope (SEM) (Phenom Corporation, Rotterdam, The Netherlands). The colors were described based on the color-codes by Rayner (1970) [38]. The following abbreviations were used: KOH = 10% potassium hydroxide, n = number of measuring objects, M = average of sizes of all measuring objects.

### 2.2. DNA Extraction and Sequencing

Total genomic DNA was extracted from fresh stromata using a rapid plant genome extraction kit (Aidlab Biotechnologies, Beijing, China) following the manufacturer’s instructions. Four loci, including nrITS, nrLSU, RPB2, and beta-tubulin (TUB), were amplified and sequenced using primers pairs ITS4/ITS5 [39], LR0R/LR5 [40], fRPB2-7CR/fRPB2-5F [41], and T1/T22 [42], respectively. The PCR procedures for ITS, LSU, RPB2 and beta-tubulin followed Ma et al. (2022) [35] in the phylogenetic analyses. Purification and sequencing were performed by the Beijing Genomics Institute (Shenzhen, China), and newly generated sequences were deposited in GenBank.

### 2.3. Phylogenetic Analysis

Phylogenetic analyses for *Hypoxylon* and related genera including *Annulohypoxylon*, *Jackrogersella*, *Parahypoxylon*, *Pyrenopolyporus*, *Rhopalostroma* and *Thamnomyces* were performed with maximum likelihood (ML) and Bayesian inference (BI) analyses based on the combined ITS-nrLSU-RPB2-TUB dataset (Table 1). *Biscogniauxia nummularia* (Bull.) Kuntze and *Xylaria hypoxylon* (L.) Grev. were used as outgroups [19].

The sequences were aligned using the online MAFFT tool (http://mafft.cbrc.jp/alignment/server/, accessed on 23 August 2023), and edited using BioEdit 7.0.5.3 [43] and ClustalX 1.83 [44]. Maximum likelihood (ML) analysis was conducted by raxmlGUI 2.0 using rapid bootstrapping with 1000 replicates, and GTRGAMMA+G as a substitution model [35]. Bayesian inference (BI) analysis was implemented in MrBayes 3.2.6 [45] using jModelTest 2 to conduct model discrimination. Six simultaneous Markov chains were run for 4,000,000 generations, from which every 100th generation was sampled as a tree. Phylogenetic trees were viewed in FigTree 1.4.2.

**Table 1:** GenBank accession numbers of sequences used in phylogenetic analyses are presented.

Species Name	Specimen No.	Locality	GenBank Accession No.				References
			ITS	LSU	RPB2	β-tubulin	
*Annulohypoxylon annulatum*	CBS 140775	USA	KU604559	KY610418	KY624263	KX376353	[33,46,47]
*A*. *truncatum*	CBS 140778	USA	KX376329	KY610419	KY624277	KX376352	[33,47]
*Biscogniauxia nummularia*	MUCL 51395	France	KY610382	KY610427	KY624236	KX271241	[33]
*Hypomontagnella barbarensis*	STMA 14081	Argentina	MK131720	MK131718	MK135891	MK135893	[34]
*Hy. monticulosa*	MUCL 54604	Guiana	KY610404	KY610487	KY624305	KX271273	[33]
*Hy. submonticulosa*	CBS 115280	France	KC968923	KY610457	KY624226	KC977267	[24,33]
*Hypoxylon addis*	MUCL 52797	Ethiopia	KC968931	-	-	KC977287	[24]
*H*. *anthochroum*	YMJ 9	Mexico	JN660819	-	-	AY951703	[24]
*H*. *aveirense*	CMG 29	Portugal	MN053021	-	-	MN066636	[48]
*H. baihualingense*	FCATAS 477	China	MG490190	-	-	MH790276	[37]
*H. baruense*	UCH 9545	Panama	MN056428	-	-	MK908142	[49]
*H*. *begae*	YMJ 215	USA	JN660820	-	-	AY951704	[32]
*H*. *bellicolor*	UCH 9543	Panama	MN056425	-	-	MK908139	[49]
*H*. *brevisporum*	YMJ 36	Puerto Rico	JN660821	-	-	AY951705	[32]
*H*. *carneum*	MUCL 54177	France	KY610400	KY610480	KY624297	KX271270	[33]
*H*. *cercidicola*	CBS 119009	France	KC968908	KY610444	KY624254	KX271270	[24,33]
*H. chrysalidosporum*	FCATAS 2710	China	OL467294	OL615106	OL584222	OL584229	[35]
*H*. *crocopeplum*	CBS 119004	France	KC968907	KY610445	KY624255	KC977268	[33]
*H. cyclobalanopsidis*	FCATAS 2714	China	OL467298	OL615108	OL584225	OL584232	[35]
*H. damuense*	FCATAS 4207	China	ON075427	ON075433	ON093251	ON093245	[19]
*H*. *dieckmannii*	YMJ 89041203	China	JN979413	-	-	AY951713	[32]
***H. diperithecium***	**FCATAS 4226**	**China**	**ON178671**	**ON350864**	**ON365561**	**ON365565**	**This study**
***H. diperithecium***	**FCATAS 4323**	**China**	**ON178672**	**ON350865**	**ON365562**	**ON365566**	**This study**
*H*. *duranii*	YMJ 85	China	JN979414	-	-	AY951714	[32]
*H*. *erythrostroma*	YMJ 90080602	China	JN979416	-	-	AY951716	[32]
*H*. *eurasiaticum*	MUCL 57720	Iran	MW367851	-	MW373852	MW373861	[50]
*H*. *fendleri*	DSM 107927	USA	MK287533	MK287545	MK287558	MK287571	[51]
*H*. *ferrugineum*	CBS 141259	Austria	KX090079	-	-	KX090080	[52]
*H*. *fragiforme*	MUCL 51264	Germany	KM186294	KM186295	KM186296	KM186293	[51]
*H*. *fraxinophilum*	MUCL 54176	France	KC968938	-	-	KC977301	[24]
*H*. *fulvosulphureum*	MFLUCC 13-0589	Thailand	KP401576	-	-	KP401584	[53]
*H*. *fuscum*	CBS 113049	France	KY610401	KY610482	KY624299	KX271271	[33]
*H. gibriacense*	MUCL 52698	Germany	KC968930	-	-	-	[24]
*H. greiderae*	BRIP 72533	USA	NR 182619	OP598062	-	-	[54]
*H*. *griseobrunneum*	CBS 331.73	India	KY610402	MH872399	KY624300	KC977303	[24,33,55]
*H*. *guilanense*	MUCL 57726	Iran	MT214997	MT214992	MT212235	MT212239	[56]
*H*. *haematostroma*	MUCL 53301	Martinique	KC968911	KY610484	KY624301	KC977291	[34]
*H. hainanense*	FCATAS 2712	China	OL467296	OL616132	OL584224	OL584231	[35]
*H*. *hinnuleum*	MUCL 3621	USA	MK287537	MK287549	MK287562	MK287575	[51]
*H*. *howeanum*	MUCL 47599	Germany	AM749928	KY610448	KY624258	KC977277	[24,33,57]
*H*. *hypomiltum*	MUCL 51845	Guadeloupe	KY610403	KY610449	KY624302	KX271249	[33]
*H*. *invadens*	MUCL 51475	France	MT809133	MT809132	MT813037	MT813038	[58]
*H*. *investiens*	CBS 118183	Malaysia	KC968925	KY610450	KY624259	KC977270	[24,33]
*H*. *isabellinum*	STMA 10247	Martinique	KC968935	-	-	KC977295	[24]
*H. jaklitschii*	JF13037	Sri Lanka	KM610290	-	-	KM610304	[24]
*H. jecorinum*	YMJ 39	Mexico	JN979429	-	-	AY951731	[32]
*H. jianfengense*	FACATAS845	China	MW984546	MZ029707	MZ047260	MZ047264	[36]
*H. larissae*	FACATAS844	China	MW984548	MZ029706	MZ047258	MZ047262	[36]
*H. laschii*	MUCL 52796	Germany	JX658525	-	-	-	[59]
*H*. *lateripigmentum*	MUCL 53304	Martinique	KC968933	KY610486	KY624304	KC977290	[24,33]
*H*. *lenormandii*	CBS 135869	Cameroon	KY610390	KY610453	KY624262	KM610295	[33,60]
*H*. *liviae*	CBS 115282	Norway	NR155154	-	-	KC977265	[24]
*H*. *lividicolor*	YMJ 70	China	JN979432	-	-	AY951734	[32]
*H*. *lividipigmentum*	YMJ 233	Mexico	JN979433	-	-	AY951735	[32]
*H*. *macrosporum*	YMJ 47	Canada	JN979434	-	-	AY951736	[32]
*H. medogense*	FCATAS 4061	China	ON075425	ON075431	ON093249	ON093243	[19]
*H. munkii*	YMJ 90080403	China	JN979436	-	-	AY951738	[32]
*H*. *musceum*	MUCL 53765	Guadeloupe	KC968926	KY610488	KY624306	KC977280	[24,33]
*H*. *notatum*	YMJ 250	USA	JQ009305	-	-	AY951739	[32]
*H*. *olivaceopigmentum*	DSM 10792	USA	MK287530	MK287542	MK287555	MK287568	[51]
*H*. *perforatum*	CBS 115281	France	KY610391	KY610455	KY624224	KX271250	[33]
*H*. *petriniae*	CBS 114746	France	NR155185	KY610491	KY624279	KX271274	[33]
*H*. *pilgerianum*	STMA 13455	Martinique	KY610412	-	KY624308	KY624315	[33]
*H*. *porphyreum*	CBS 119022	France	KC968921	KY610456	KY624225	KC977264	[24,33]
*H*. *pseudofendleri*	MFLUCC 11-0639	Thailand	KU940156	KU863144	-	-	[61]
*H*. *pseudofuscum*	18264	Germany	MW367857	MW367848	MW373858	MW373867	[50]
*H*. *pulicicidum*	CBS 122622	Martinique	JX183075	KY610492	KY624280	JX183072	[33,62]
*H. rickii*	MUCL 53309	Martinique	KC968932	KY610416	KY624281	KC977288	[33]
*H. rubiginosum*	MUCL 52887	Germany	KC477232	KY610469	KY624266	KY624311	[33,63]
*H. rutilum*	YMJ 181	France	-	-	-	AY951752	[32]
*H. samuelsii*	MUCL 51843	Guadeloupe	KC968916	KY610466	KY624269	KC977286	[24,33]
*H. shearii*	YMJ 29	Mexico	EF026142	-	-	AY951753	[32]
*H. spegazzinianum*	STMA 14082	Argentina	KU604573	-	-	KU604582	[64]
*H. sporistriatatunicum*	UCH 9542	Panama	MN056426	-	-	MK908140	[49]
*H. subgilvum*	YMJ 88113007	China	JQ009315	-	-	AY951755	[32]
*H. sublenormandii*	JF 13026	Sri Lanka	KM610291	-	-	KM610303	[60]
*H. teeravasati*	PUFD4	India	KY863509	MF385274	MG986895	MG986894	[65]
*H. texense*	DSM 107933	USA	MK287536	MK287548	MK287561	MK287574	[51]
***H. tibeticum***	**FCATAS4022**	**China**	**OR654146**	**OR654303**	**ON254302**	**ON230084**	**This study**
***H. tibeticum***	**FCATAS4371**	**China**	**OR654263**	**OR654304**	**QQ303928**	**QQ303964**	**This study**
***H. tibeticum***	**FCATAS4212**	**China**	**OR654264**	**OR654305**	**ON254308**	**ON254275**	**This study**
***H. tibeticum***	**FCATAS4373**	**China**	**OR654265**	**OR654306**	**QQ303933**	**QQ303965**	**This study**
*H. ticinense*	CBS 115271	France	JQ009317	KY610471	KY624272	AY951757	[32,33]
*H. trugodes*	MUCL 54794	Sri Lanka	KF234422	NG066380	KY624282	KF300548	[24,33]
*H. ulmophilum*	YMJ 350	Russia	JQ009320	-	-	AY951760	[32]
*H. vinosopulvinatum*	YMJ 90080707	China	JQ009321	-	-	AY951761	[32]
*H. vogesiacum*	CBS 115273	France	KC968920	KY610417	KY624283	KX271275	[33]
*H. wujiangense*	GMBC0213	China	MT568854	MT568853	MT585802	MT572481	[66]
*H. wuzhishanense*	FCATAS 2708	China	OL467292	OL615104	OL584220	OL584227	[35]
*H. zangii*	FCATAS 6092	China	OQ316425	OQ348528	OQ303910	OQ303948	[19]
*Jackrogersella cohaerens*	CBS 119126	Germany	KY610396	KY610497	KY624270	KY624314	[33]
*J. multiformis*	CBS 119016	Germany	KC477234	KY610473	KY624290	KX271262	[24,33]
*Parahypoxylon papillatum*	ATCC 58729	USA	NR155153	KY610454	KY624223	KC977258	[24,33]
*Pyrenopolyporus hunteri*	MUCL 52673	Ivory Coast	KY610421	KY610472	KY624309	KU159530	[33,47]
*Py.laminosus*	MUCL 53305	Martinique	KC968934	KY610485	KY624303	KC977292	[24,33]
*Py. nicaraguensis*	CBS 117739	Burkina Faso	AM749922	KY610489	KY624307	KC977272	[24,33,57]
*Rhopalostroma angolense*	CBS 126414	Ivory Coast	KY610420	KY610459	KY624228	KX271277	[33]
*Thamnomyces dendroidea*	CBS 123578	FrenchGuiana	FN428831	KY610467	KY624232	KY624313	[33,67]
*Xylaria hypoxylon*	CBS 122620	Sweden	KY610407	KY610495	KY624231	KX271279	[33]

Species in bold were derived from this study. “-” are not available.

## 3. Results

### 3.1. Phylogenetic Analysis

The phylogeny of *Hypoxylon* and related genera based on a combined ITS-nrLSU-RPB2-TUB dataset included 98 ITS, 64 nrLSU, 65 RPB2 and 95 TUB sequences from 97 specimens representing 93 taxa. There were 2852 character positions for ITS alignment, 3462 character positions for LSU alignment, 1288 character positions for RPB2 alignment, and 2225 character positions for TUB alignment. The dataset of four DNA loci had an aligned length of 3538 characters, of which 1520 characters were parsimony informative.

**Figure 1:**
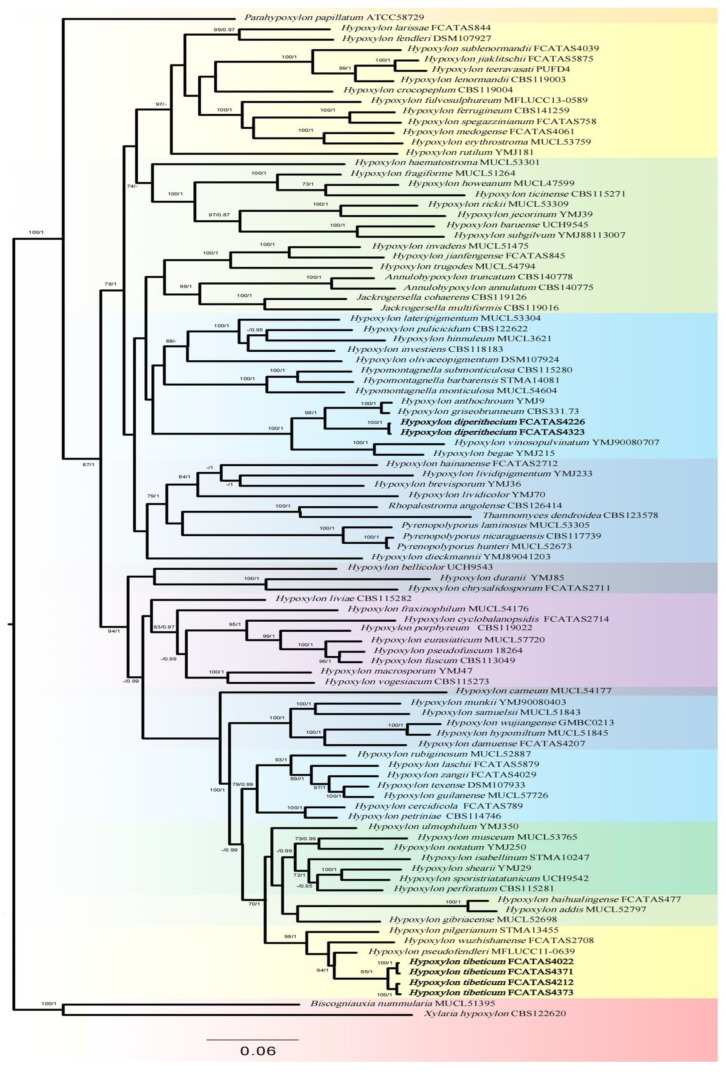
BI phylogenetic tree of the genus *Hypoxylon* inferred from multi-gene alignment of ITS-LSU-RPB2-TUB. ML bootstrap support (BS) ≥ 70% and Bayesian posterior probabilities (PP) ≥ 0.95 are given at the nodes in this order. New species in this study are indicated in bold.

The topologies from BI and ML analyses are highly similar; the BI tree is shown in this study. Branches that received bootstrap support for maximum likelihood (ML) higher than or equal to 70% (ML-BS) and Bayesian posterior probabilities (BPP) higher than or equal to 0.95 (BPP) were showed in topologies. In phylogenetic analysis, the two new species were clearly separated from other sampled species of *Hypoxylon*. The two strains of *H. diperithecium* were closely related to *H. anthochroum* Berk. & Broome and *H. griseobrunneum* (B.S. Mehrotra) J. Fourn., Kuhnert & M. Stadler with high support (BS = 98, PP = 1.00, Figure 1), and four strains of *H. tibeticum* clustered with *H. pseudofendleri* D.Q. Dai, K.D. Hyde with high support (BS = 94, PP = 1.0, Figure 1).

### 3.2. Taxonomy

**Figure 2:**
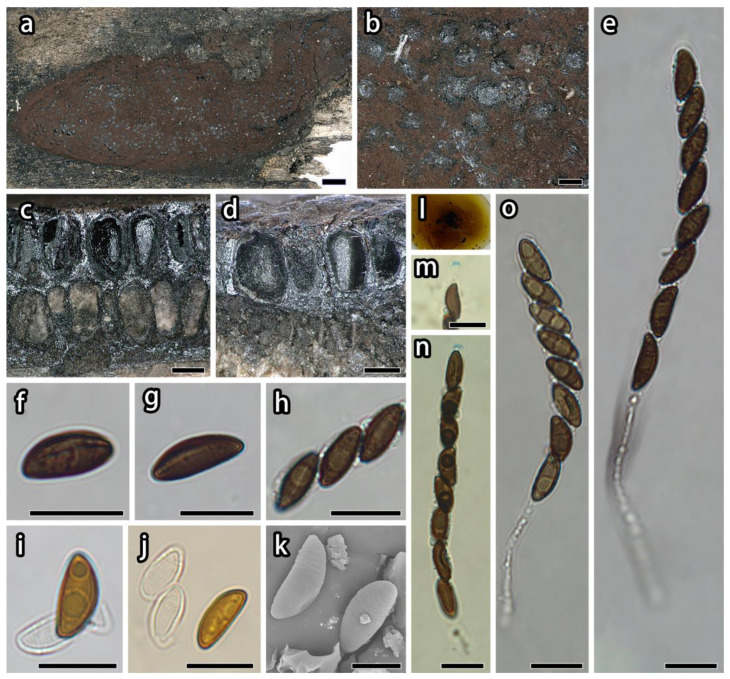
*Hypoxylon diperithecium* (holotype, FCATAS 4226). (**a**,**b**). Stromata; (**c**,**d**). Stroma in vertical section showing the perithecia and tissue below the perithecial layer; (**e**,**o**). Ascus in water; (**f**,**g**). Ascospore in water; (**h**). Ascospores and germ slit; (**i**,**j**). Ascospore in 10% KOH; (**k**). Ascospores under SEM; (**l**). KOH-extractable pigments; (**m**). Apical apparatus; (**n**). Ascus in Melzer’s reagent. Scale bars: (**a**) = 1 mm; (**b**–**d**) = 200 μm; (**e**–**j**,**m**–**o**) = 10 μm; (**k**) = 5 μm.

***Hypoxylon diperithecium*** Hai X. Ma, Z.K. Song & A.H. Zhu, sp. nov., Figure 2.

MycoBank: MB850560

**Diagnosis.** Differs from *H. griseobrunneum* in its two layers of perithecia, smaller perithecia and asci with shorter stipes. Differs from *H. subgilvum* in its perithecial layer and color of KOH-extractable pigments and ascospores.

**Etymology.** The epithet *diperithecium* (Lat.) refers to the species has bistratal perithecia.

**Holotype.** China: Tibet Autonomous Region, Motuo County, Damu Township, Kabu Village, 29°38′42″ N, 95°37′44″ E, alt. 1280 m, saprobic on the bark of dead wood, 2 October 2021, Haixia Ma & Zikun Song, FCATAS 4226 (XZ226).

**Teleomorph.** Stromata pulvinate, 1.4–5 × 0.4–1.3 cm × 0.8–1.2 mm thick; with inconspicuous to conspicuous perithecial mounds; surface livid purple (81) to bay (6), exposing black subsurface layer when colored coating worn off; with purple-brown granules immediately beneath the surface and between perithecia; yielding citrine (13) to rust (39) KOH-extractable pigments; tissue below the perithecial layer dark brown, 0.1–0.7 mm thick. Perithecia ovoid to tubular, bilayer, black, 0.1–0.3 × 0.25–0.45 mm. Ostioles opening higher than the stromatal surface. Asci cylindrical with eight obliquely uniseriate ascospores, 78–139 µm total length, the spore-bearing portion 56–73 × 5.2–7.6 µm, and stipes 23–77 µm long, with amyloid apical apparatus bluing in Melzer’s reagent, discoid, 0.7–0.8 × 1.9–2.1 µm. Ascospores light brown to brown, unicellular, ellipsoid-inequilateral, with narrowly rounded ends, 9.2–11.6 × 4–5.7 µm (n = 60, M = 10.2 × 4.8 µm), with straight spore-length germ slit on the convex side; perispore dehiscent in 10% KOH, with conspicuous coil-like ornamentation in SEM; epispore smooth.

**Additional specimens examined.** China: Tibet Autonomous Region, Motuo County, Damu Township, Kabu Village, 29°37′45″ N, 95°37′50″ E, alt. 1300 m, saprobic on the bark of dead wood, 2 October 2021, Haixia Ma & Zikun Song, Col. XZ323 (FCATAS 4323).

**Note.** Some stromata of *Hypoxylon diperithecium* have two layers of perithecia visible, and the upper and the lower may be same species according to morphology of ascospore and perithecia; this feature is similar to *H. subgilvum* Berk. & Broome. *Hypoxylon subgilvum* has three stromatal layers with the basal layer an effete *Biscogniauxia*, and other two layers are considered the same species [23,68]. Morphologically, *H. subgilvum* can be distinguished from *H. diperithecium* by its orange red stromatal granules, KOH-extractable pigments orange, and brown to dark brown ascospores [23]. Moreover, molecular evidence supported *H. diperithecium* as a distinct species from *H. subgilvum* (Figure 1).

Although *H. anthochroum* and *H. griseobrunneum* were grouped with *H. diperithecium* (Figure 1), they differ from the new species proposed here because the former has only one layer of perithecia instead of two layers and has dull reddish brown or blackish granules immediately beneath surface and between perithecia, yielding isabelline (65), olivaceous (48), gray olivaceous (107), greenish olivaceous (90), or amber (47) KOH-extractable pigments [23]. While some stromata of *H. griseobrunneum* tend to develop multiple perithecial layers, it can be distinguished from *H. diperithecium* by having larger perithecia, with KOH-extractable pigments Fawn (87), and longer stipes of asci (76–86 µm) [24]. Therefore, *H. diperithecium* is proposed as a new species.

**Figure 3:**
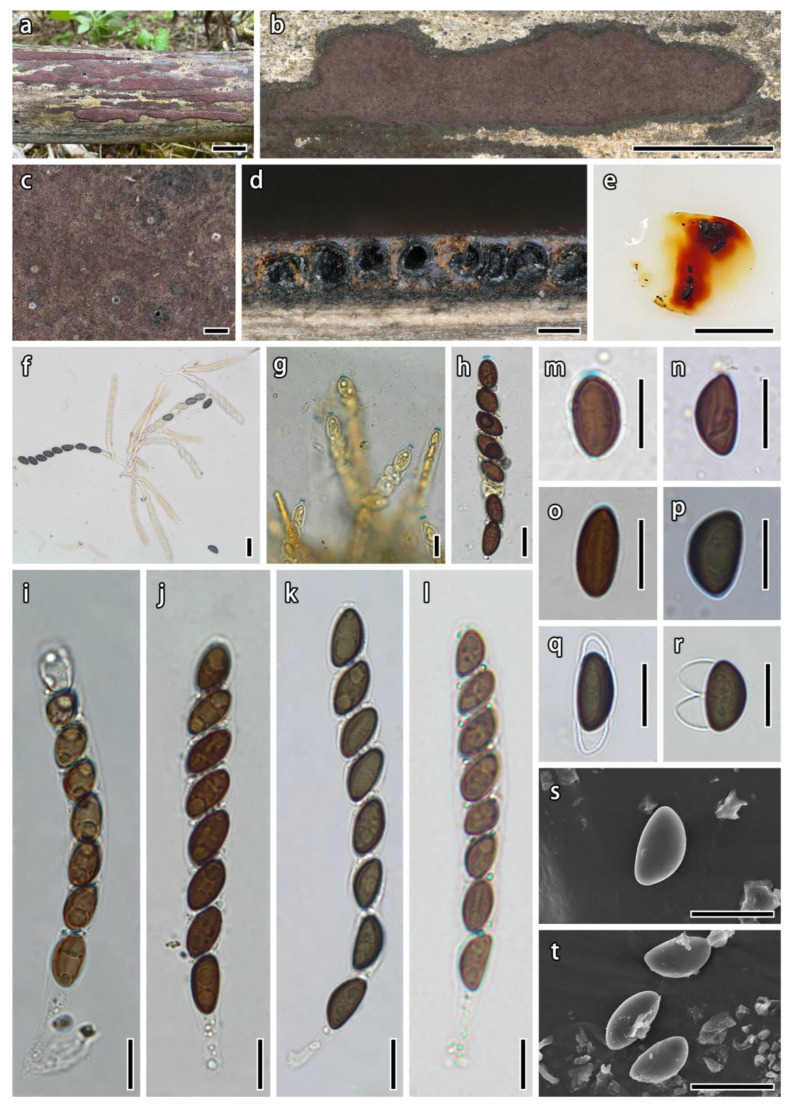
*Hypoxylon tibeticum* (holotype, FCATAS 4226). (**a**,**b**). Stromata; (**c**). Stromatal surface; (**d**). Stroma in vertical section showing the perithecia and tissue below the perithecial layer; (**e**). KOH-extractable pigments; (**f**). Ascus in water; (**g**,**h**). Ascus and apical apparatus in Melzer’s reagent; (**i**–**l**). Ascus in water; (**m**–**p**). Ascospore in water; (**q**,**r**). Ascospores in 10% KOH; (**s**,**t**). Ascospores under SEM. Scale bars: (**a**,**b**,**e**) = 1 cm; (**c**,**d**) = 200 μm; (**f**) = 20 μm; (**g**–**t**) = 10 μm.

***Hypoxylon tibeticum*** Hai X. Ma, Z.K. Song & A.H. Zhu, sp. nov., Figure 3.

MycoBank: MB850558

**Diagnosis.** Differs from *H pseudofendler* in its smaller perithecia and slightly larger ascospores. Differs from *H. wuzhishanense* in its brown vinaceous stromatal surface with orange granules between perithecia and perispore dehiscent in KOH. Differs from *H. pilgerianum* in its larger ascospores.

**Etymology.** The epithet *tibeticum* (Lat.) refers to the locality (Tibet Autonomous Region) of the type specimens.

**Holotype.** China: Tibet Autonomous Region, Motuo County, Damu Township, Kabu Village, the large bend of Linduo, 29°27′51″ N, 95°26′39″ E, alt. 781 m, saprobic on the stems of dead bamboo, 24 September 2021, Haixia Ma & Zikun Song, FCATAS 4022 (XZ22).

**Teleomorph.** Stromata effused-pulvinate, applanate, 1.4–11.1 × 0.2–1.5 cm × 0.2–0.35 mm thick, irregularly elongate, often coalescent; surface brown vinaceous (84) or dark vinaceous (85), pruinose, with inconspicuous to slightly conspicuous perithecial mounds; with orange granules immediately beneath the surface and between perithecia; yielding orange (7) KOH-extractable pigments; the tissue beneath the perithecia dark brown, 0.05–0.15 mm thick. Perithecia spherical, black, 0.1–0.23 mm diam. Ostioles umbilicate, opening lower than the stromatal surface, mostly fringed with white material forming a disc. Asci cylindrical, with eight obliquely uniseriate ascospores, 75–101 µm total length, the spore-bearing portion 64–91 × 7.8–11.5 µm, and stipes 9–17 µm long, with amyloid apical apparatus bluing in Melzer’s reagent, discoid, 0.89–1.54 × 2.1–2.95 µm. Ascospores brown, unicellular, ellipsoid-inequilateral, with narrowly to broad rounded ends, 9.8–13 × 5.1–6.9 µm (n = 60, M = 11.34 × 6.21 µm), with straight spore-length germ slit on the convex side; perispore dehiscent in 10% KOH, with faint inconspicuous coil-like ornamentation in SEM; epispore smooth.

**Additional specimens examined.** China: Tibet Autonomous Region, Motuo County, Damu Township, Kabu Village, the large bend of Linduo, 29°27′51″ N, 95°26′39″ E, alt. 780 m, saprobic on the stems of dead bamboo, 24 September 2021, Haixia Ma & Zikun Song, FCATAS 4371 (XZ324); Kabu Village, 29°37′45″ N, 95°37′50″ E, alt. 1280 m, saprobic on dead bamboo, 2 October 2021, Haixia Ma & Zikun Song, FCATAS4212 (XZ212), FCATAC4373 (XZ326).

**Note.** Based on the phylogenetic analyses, four species of *Hypoxylon* growing on dead bamboo culms grouped together (Figure 1), including *H. pilgerianum* Henn., *H. pseudofendleri* D.Q. Dai & K.D. Hyde, *H. wuzhishanense* Hai X. Ma & Z.K. Song, and the new species *H. tibeticum*.

In the phylogenetic tree (Figure 1), *H. tibeticum* is the sister species of *H. pseudofendleri* from Thailand with strong support values (BS = 94, PP = 1). Morphologically, both *H. tibeticum* and *H. pseudofendleri* have effused-pulvinate and purplish-brown stromata, with orange granules beneath the surface and between perithecia. However, *H pseudofendleri* differs in its larger perithecia (0.5–0.85 × 0.35–0.5 mm), ostioles slightly higher than the stromatal surface, and slightly smaller ascospores (9–11.5 × 4.5–6.5 µm, M = 10.2 × 5.7 µm) [61]. *Hypoxylon wuzhishanense* from Hainan tropical rainforest of China has similar stromatal morphology and ascospores size, but it has rust (39), livid purple (81) to dark brick (60) stromatal surface, with yellowish-brown granules beneath the surface and between perithecia, and most of perispore indehiscent in 10% KOH [35]. *Hypoxylon pilgerianum* was first described from Brazil on culms of *Chusquea* [69]; subsequently, many specimens on culms of dead bamboo were found from China, Madagascar, Malaysia, Papua New Guinea, and Trinidad [23,68]. *Hypoxylon pilgerianum* s. Ju & Rogers is similar to *H. tibeticum* in stromatal morphology, but it differs in having shorter [8.5–12 (–13.5) μm] and narrower ascospores [4–5 (–5.5) μm] [23]. Moreover, the phylogenetic analyses (Figure 1) showed that they are different species.

**Dichotomous key to *Hypoxylon* species from China**

1. Stromata on bamboo ............................................................................................................ 2

1. Stromata on dicot wood ...................................................................................................... 4

2. Most perispore indehiscent in 10% KOH .............................................. ***H. wuzhishanense***

2. Perispore dehiscent in 10% KOH ....................................................................................... 3

3. KOH-extractable pigments ochreous (44), honey (64) or amber (47); ascospores 8.5–12 (–13.5) × 4– 5 (–5.5) µm ............................................................................. ***H. pilgerianum***

3. KOH-extractable pigments orange (7); ascospores 9.8–13 × 5.1–6.9 µ....... ***H. tibeticum***

4. Stromatal surface dark cyan blue or olivaceous ............................................................... 5

4. Stromatal surface other colors.............................................................................................. 6

5. Stromatal surface dark cyan blue; ascospores 11.5–13.5 × 5–6 µm..............***H. cyanescens***

5. Stromatal surface olivaceous or isabelline; ascospores 9–13 × (4–) 4.5–6 µm .........................................................................................................................***H. musceum***

6. Ascospores equilateral or nearly equilateral ......................................................................7

6. Ascospores inequilateral ......................................................................................................16

7. Ostioles higher than the stromatal surface ........................................................................8

7. Ostioles lower than the stromatal surface .........................................................................9

8. Stromata glomerate to pulvinate, with very conspicuous perithecial mounds; KOH-extractable pigments isabelline (65) or hazel (88) ...........................................***H. croceum***

8. Stromata pulvinate, with inconspicuous perithecial mounds; KOH-extractable

pigments brick (59) ........................................................................................***H. parksianum***

9. Perispore dehiscent in 10% KOH............................................................... ***H. hypomiltum***

9. Perispore indehiscent in 10% KOH.....................................................................................10

10. Perithecia tubular to long tubular........................................................................................ 11

10. Perithecia obovoid .................................................................................................................13

11. Stromatal surface fulvous (43), rust (39), sinna (8), ochreous (44), or apricot (42); KOH-extractable pigments orange (7) ...............................................................***H. cinnabarinum***

11. Stromatal surface sepia (63) or chestnut (40) ......................................................................12

12. KOH-extractable pigments greenish yellow (16), dull green (70), or dark green (21);

ascospores 6.5–9.5 (–10) × 3–4.5 µm ..................................................................***H. investiens***

12. KOH-extractable pigments livid violet (79), violaceous gray (113), or violet slate (99);

ascospores (10.5–) 11–16 × (4.5–) 5–6.5 µm .................................................***H. sclerophaeum***

13. Without apparent KOH-extractable pigments or dilute grayish sepia ..........................14

13. With KOH-extractable pigments .........................................................................................15

14. Without apparent KOH-extractable pigments or dilute grayish sepia (106); ascospores

6.5–10 (–11) × (3–) 3.5–4 µm ......................................................................... ***H. dieckmannii***

14. Without apparent KOH-extractable pigments; ascospores (9.5–) 10.5–11.5 (–12.5) ×

4.5–6 µm .......................................................................................................... ***H. yunnanense***

15. KOH-extractable pigments olivaceous (48), greenish olivaceous (90), gray olivaceous

(127), or olivaceous gray (121); ascospores (11.5–) 12–15 (–16) × 5.5–7 µm ......................

.....................................................................................................................***H. fuscopurpureum***

15. KOH-extractable pigments hazel (88); ascospores 7–8.5 × 4–4.5 µm.......... ***H. gilbertsonii***

16. Stromata hemispherical to spherical .................................................................................17

16. Stromata pulvinate to effused-pulvinate...........................................................................23

17. Ascospore length up to 20 µm ..........................................................................................18

17. Ascospore length less than 20 µm.....................................................................................19

18. Ascospores 18–28 × 6–10 µm........................................................................... ***H. apiculatum***

18. Ascospores 8–20 × 4–8 µm....................................................................................... ***H. fuccum***

19. Perithecia tubular .................................................................................................................20

19. Perithecia spherical to obovoid ..........................................................................................21

20. Stromata with orange red granules, with KOH-extractable pigments orange (7) or

scarlet (5); ascospores 13.5–18 (–19) × 7–8 (–8.5) µm............................. ***H. haematostroma***

20. Stromata with dark reddish brown or blackish granules, with KOH-extractable

pigments olivaceous (48), greenish olivaceous (90), isabelline (65), or dull green (70);

ascospores 8.5–18.5 × 4.5–8 (–8.5) µm........................................................ ***H. placentiforme***

21. KOH-extractable pigments amber (47) with greenish yellow (16) tone, or greenish

yellow (16) with citrine (13) tone; ascospores (8–) 9–12 (–13) × 4–6 µm...... ***H. perforatum***

21. KOH-extractable pigments orange (7) ..............................................................................22

22. Ascospores (10.5–) 11–15 × 5–6.5 (–7) µm, with straight germ slit................***H. fragiforme***

22. Ascospores 7–9.5 (–10) × 3–4.5 µm, with slight sigmoid germ slit ............***H. howeianum***

23. Ostioles at the same level or higher than the stromatal surface ....................................24

23. Ostioles lower than the stromatal surface .........................................................................29

24. Perithecia tubular, ascospores 6–7.5 × 3–3.5 µm.................................... ***H. lienhwacheense***

24. Perithecia spherical to obovoid............................................................................................25

25. KOH-extractable pigments orange (7), scarlet (5) or amber (47) .....................................26

25. KOH-extractable pigments with other colors ....................................................................28

26. Stromata with red or scarlet granules; ascospores 7.5–9.5 × 3.5–4.5 µm........... ***H. rutilum***

26. Stromata with orange granules ...........................................................................................27

27. Ascospores 8–10 × 3.5–4.5 µm.................................................................................. ***H. laschii***

27. Ascospores 9.9–12.8 × 4.6–7 µm....................................................................... ***H. medogense***

28. KOH-extractable pigments hazel (88), sienna (8), cinnamon (62), fulvous (43), umber

(9), or ochreous (44); ascospores 9.5–15 (–16) × 4–6.5 (–7) µm ................... ***H. lenormandii***

28. KOH-extractable pigments pale vinaceous (85) to livid vinaceous (83) and vinaceous

purple (101); ascospores 6.1–9.6 × 3.2–5 µm .................................................. ***H. hainanense***

29. Without apparent KOH-extractable pigments; ascospores (12–) 13–16 × 5–6 µm ..........

................................................................................................................ ***H. kretzschmarioides***

29. With KOH-extractable pigments ........................................................................................30

30. Most ascospore length less than 8 µm ..............................................................................31

30. Most ascospore length more than 8 µm ...........................................................................33

31. KOH-extractable pigments olivaceous gray (12), greenish olivaceous (90), or gray

olivaceous (107); ascospores 5.5–8 × 2.5–3.5 µm ........................................ ***H. brevisporum***

31. KOH-extractable pigments orange.......................................................................................32

32. Perithecia obovoid to tubular; ascospores (4.5–) 5–7 × 2.5–3.5 µm ....................................

.............................................................................................. ***H. subgilcum*** var. ***microsporum***

32. Perithecia spherical to ovoid; ascospores 6.5–8.5 × 4–5 µm .......................... ***H. hubeiense***

33. Asci with apical apparatus highly reduced or lacking, not bluing in Melzer’s reagent. ...

...................................................................................................................................................34

33. Asci with apical apparatus bluing in Melzer’s reagent.....................................................39

34. KOH-extractable pigments orange tone..............................................................................35

34. KOH-extractable pigments other colors..............................................................................37

35. Ascospores with inconspicuous coil-like ornamentation, (9–) 9.5–12 × 5–6 µm................

..............................................................................................................................***H. cercidicola***

35. Ascospores with conspicuous coil-like ornamentation.....................................................36

36. KOH-extractable pigments orange (7), sienna (8), or amber (47); ascospores 9.2–15.6 × 5.5–7.5 µm, with spore-length straight germ slit........................................***H. baihualingense***

36. KOH-extractable pigments luteous (12); ascospores 12–14 × 5.5–6.5 (–7) µm....***H. shearii***

37. Ascospores with inconspicuous coil-like ornamentation, (11–) 12–16 × (5.5–) 6–7.5 µm....

..................................................................................................................................***H. notatum***

37. Ascospores with conspicuous coil-like ornamentation...................................................38

38. Ascospores 8–10.6 (–11.1) × 4.1–6.3 (–7.1) µm, with conspicuously straight spore-length

germ slit..................................................................................................***H. chrysalidosporum***

38. Ascospores 11–15.2 × 5.1–7 µm, with more sigmoid to less straight spore-length germ

slit............................................................................................................***H. cyclobalanopsidis***

39. Ascospores with conspicuous coil-like ornamentation....................................................40

39. Ascospores smooth or with inconspicuous coil-like ornamentation..............................44

40. Most perispore indehiscent in 10% KOH; ascospores 8.2–10.5 × 4.1–5.5 µm. ....................

....................................................................................................................................***H. damuense***

40. Perispore dehiscent in 10% KOH..........................................................................................41

41. Ascospores with straight germ slit.......................................................................................42

41. Ascospores with straight to slightly sigmoid germ slit......................................................43

42. Perithecia bilayer; ascospores 9.2–11.6 × 4–5.7 µm..................................***H. deperithecium***

42. Perithecia monolayer; ascospores 10.3–13.6 × (4.2–) 4.7–6.1 µm................***H. jianfengense***

43. KOH-extractable pigments orange (7) or scarlet (5); ascospores (9) 9.5–12 × 4.5–5 µm.......

.....................................................................................................................................***H. retpela***

43. KOH-extractable pigments isabelline (65) or amber (47); ascospores 9.5–13 (–14.5) ×

4.5–6.5 µm..................................................................................................................***H. duranii***

44. Ascospore length up to 15 µm ...........................................................................................45

44. Ascospore length less than 15 µm .....................................................................................46

45. Stromatal surface cinnamon (62), fulvous (43), apricot (42), sienna (8), rust (39), or bay

(6); ascospores (9–) 9.5–15 (–17.5) × 4–7 (–7.5) µm.......................................***H. crocopeplum***

45. Stromatal surface rust (39), sienna (8), fulvous (43), or bay (6); ascospores 15.5–22.9 (–

23.6) × 7.3–10.6 µm...................................................................................................***H. larissae***

46. Ascospores with sigmoid germ slit......................................................................................47

46. Ascospores with straight, straight or slightly sigmoid germ slit.....................................48

47. KOH-extractable pigments orange (7); ascospores (8–) 9–12 × 4–5.5 µm..........***H. fendleri***

47. KOH-extractable pigments vinaceous purple (101); ascospores 9.5–12.5 × 5–6 µm...........

.................................................................................................................................***H. fuscoides***

48. Ascospores with straight germ slit.......................................................................................49

48. Ascospores with straight to slightly sigmoid germ slit......................................................53

49. KOH-extractable pigments orange tone .............................................................................50

49. KOH-extractable pigments other colors .............................................................................51

50. Stromata with orange granules; ascospores (10–) 10.5–11.5 (–12.5) × 5–6.5 µm..............

.......................................................................................................................................***H. dengii***

50. Stromata with yellowish brown or brown granules; ascospores (8–) 9–12 × 4–5.5 µm.......

...........................................................................................................................***H. rubiginosum***

51. Perithecia obovoid to tubular; ascospores 8–11 × 3.5–4.5 µm ...........................***H. trugodes***

51. Perithecia spherical, ovoid to obovoid................................................................................52

52. Stromatal surface brown vinaceous; ascospores 11–13 × 5–6 µm ...***H. vinosopulvinatum***

52. Stromatal surface livid red and vinaceous; ascospores 10.9–14.6 × 4.8–6.4 µm ..***H. zangii***

53. KOH-extractable pigments orange ......................................................................................54

53. KOH-extractable pigments other colors .............................................................................55

54. Asci with apical apparatus bluing to faintly bluing in Melzer’s iodine reagent, 0.3–1 µm high × 1.5–2.2 µm broad; ascospores 7–11 × 3.5–5 µm....................................***H. subgilvum***

54. Asci with apical apparatus bluing in Melzer’s iodine ragent, 0.2–0.5 µm high × 1–1.5 µm broad; ascospores 8–9.5 (–11) × 4–5 µm.........................................................***H. jecorinum***

55. Perithecia tubular; ascospores 11–12.5 × 4.5–5 µm........................................***H. lividicolor***

55. Perithecia subglobose or obovoid to tubular......................................................................56

56. Perithecia obovoid to tubular; ascospores 8.5–13.5 × 4–6 µm...................***H. anthochroum***

56. Perithecia subglobose; ascospores 8.5–10 × 4.5–6 µm................................***H. wujiangensis***

## 4. Discussion

Currently, the genus *Hypoxylon* is still considered a paraphyletic group in Hypoxylaceae based on a single-region (ITS sequences) or multi-locus phylogeny involving both protein-coding and rDNA genes [33,70,71,72]. In this study, two species of *Hypoxylon* from Tibet of China, *H. diperithecium* and *H. tibeticum*, are proposed as new species based on morphological features and multi-gene (ITS-LSU-RPB2-TUB) phylogenetic analyses. Fifty-five species of *Hypoxylon* have been reported and described in China [19,35,36,66,73,74], and this study expanded the numbers of *Hypoxylon* species to 57 around China. However, studies in China are still few and the relationships amongst *Hypoxylon* species remain unresolved. Therefore, more comprehensive studies on the diversity, phylogeny, and evolution of the genus *Hypoxylon* depend on more collections and data from poorly sampled areas. With the in-depth investigation of *Hypoxylon* in Tibet, an increasing number of new species and new records will be discovered, and the species diversity will be richer.

## Data Availability

All sequences newly generated were deposited in GenBank (https://www.ncbi.nlm.nih.gov/genbank/ (accessed on 16 October 2023); Table 1). All new taxa were deposited in MycoBank (https://www.mycobank.org/ (accessed on 18 October 2023); MycoBank identifiers follow new taxa).
